# Influence of Time-Pickoff Circuit Parameters on LiDAR Range Precision

**DOI:** 10.3390/s17102369

**Published:** 2017-10-17

**Authors:** Xiaolu Li, Hongming Wang, Bingwei Yang, Jiayue Huyan, Lijun Xu

**Affiliations:** 1 School of Instrument Science and Opto-Electronic Engineering, Beihang University, Beijing 100191, China; wanghm16@163.com (H.W.); bingweiyang1966@163.com (B.Y.); hyjy_buaa@163.com (J.H.); lijunxu@buaa.edu.cn (L.X.); 2 State Key Laboratory of Inertial Science and Technology, School of Instrumentation Science and Opto-electronic Engineering, Beihang University, Beijing 100191, China

**Keywords:** time-pickoff circuit, automatic gain control, timing discriminators, Monte Carlo simulations, attenuation fraction, delay time

## Abstract

A pulsed time-of-flight (TOF) measurement-based Light Detection and Ranging (LiDAR) system is more effective for medium-long range distances. As a key ranging unit, a time-pickoff circuit based on automatic gain control (AGC) and constant fraction discriminator (CFD) is designed to reduce the walk error and the timing jitter for obtaining the accurate time interval. Compared with Cramer–Rao lower bound (CRLB) and the estimation of the timing jitter, four parameters-based Monte Carlo simulations are established to show how the range precision is influenced by the parameters, including pulse amplitude, pulse width, attenuation fraction and delay time of the CFD. Experiments were carried out to verify the relationship between the range precision and three of the parameters, exclusing pulse width. It can be concluded that two parameters of the ranging circuit (attenuation fraction and delay time) were selected according to the ranging performance of the minimum pulse amplitude. The attenuation fraction should be selected in the range from 0.2 to 0.6 to achieve high range precision. The selection criterion of the time-pickoff circuit parameters is helpful for the ranging circuit design of TOF LiDAR system.

## 1. Introduction

Light Detection and Ranging (LiDAR) is a remote sensing method that uses a laser beam to measure target ranges. There are several ranging methods can be applied in LiDAR, such as triangulation, interferometry, pulsed time-of-flight (TOF) measurement, and phase-shift measurement [1]. Triangulation, interferometry and phase-shift measurement have higher measurement accuracy, which can reach millimeter or even micron level [2], but the pulsed TOF measurement is more effective over medium-long range distances [3]. In a pulsed TOF LiDAR system, a time-pickoff circuit is an essential component to measure the time interval between the emitted pulse and the returned pulse, so that the parameter determination of the time-pickoff circuit is a crucial issue for designing and implementing a TOF LiDAR system.

Due to atmospheric absorption and scattering, uncertain reflectivity of target surfaces, noise interference and other factors, the measured time interval is usually not fixed, interfering with the LiDAR measurement accuracy and precision [4]. The main inaccuracy sources in the time-pickoff circuit consist of walk error, timing jitter, drift and nonlinearity [5]. Walk error is defined as the timing error resulting from the variation of the amplitude and shape of the input pulses, which is the main systematic error of LiDAR [5]. Timing jitter refers to the timing deviation caused by noise such as statistical fluctuations existing in the shape of the detected pulses, which is the largest random error [6]. Drift and nonlinearity can be reduced to a negligible level by periodic calibration [6]. Therefore, reducing the walk error and timing jitter is of great concern so that the time-pickoff circuit can be designed to obtain an accurate time interval.

Theoretically speaking, constant fraction determinators (CFDs) are widely used in time-pickoff circuits to eliminate the walk error due to their insensitivity to pulse amplitude [7]. However, there are two imperfections in CFDs that need to be overcome in real applications. Firstly, the response time of comparator of a CFD is limited by the overdrive of the comparator and the slope of the input pulses, which can generate an extra-walk-error [8]. Secondly, timing jitter in a CFD is influenced by the increase of the pulse amplitudes [6]. Thus, the walk error and the timing jitter are not actually reduced adequately if only a CFD is used in the time-pickoff circuit, especially when the input pulse amplitude varies over a wide dynamic range [7]. This paper proposes a ranging circuit parameter determination method for improving the measurement accuracy and precision of LiDAR systems.

There are three types of methods to study the relationship between the parameters determination (circuit parameter and pulse parameter) and the range precision, which are theoretical analysis methods, simulation methods and empirical methods. For the theoretical analysis method, Cramer–Rao lower bound (CRLB) on range estimate and the probability density distribution of range data have been studied, which can be utilized as a limit for the range estimate to describe the range properties [9,10]. For simulation methods, Monte-Carlo simulation is the most common model, which is utilized to evaluate the timing performance of a CFD as an effective way to optimize scintillation-detector timing systems [11]. From 2010 to 2016, Monte-Carlo simulations combining CRLB were used to evaluate the influenced on range precision of different parameters such as the amplitude, pulse width and bias and tilt angle of flat surfaces [12,13]. For empirical methods, several researchers have utilized experiments to illustrate the influence on range precision of pulse density, noise and atmosphere, and have proposed methods to improve the range precision [14,15,16]. These simulations provide a diversity of simulated data for the development of application that should be optimized for a real system [14]. In general, simulation methods and empirical method lack in-depth analysis of how the range precision is influenced by the circuit parameters and pulse parameters and thus they cannot guild the design and optimization of the time-pickoff circuit based on AGC and timing discriminators. In this paper, we simulated the range measurement by using Monte-Carlo simulation and implemented a series of experiments for adjusting the parameters to optimize the output of the time-pickoff circuit. The resulting analysis is based on a combination of simulation and circuit design.

The main contributes of this study may be stated as follows: first, a high-performance time-pickoff circuit for a LiDAR system was put forward based on AGC and timing discriminators, including CFD, which can effectively reduce walk error and timing jitter. Second, the related parameters including the pulse amplitude, the pulse width, the attenuation factor and the delay time involved in time-pickoff circuit are simulated for discussing their influence on the range precision. Finally, a series of experiments were performed to verify the way the range precision is influenced by the ranging circuit parameters.

## 2. System Design

### 2.1. Lab-Build LiDAR System

**Figure 1:**
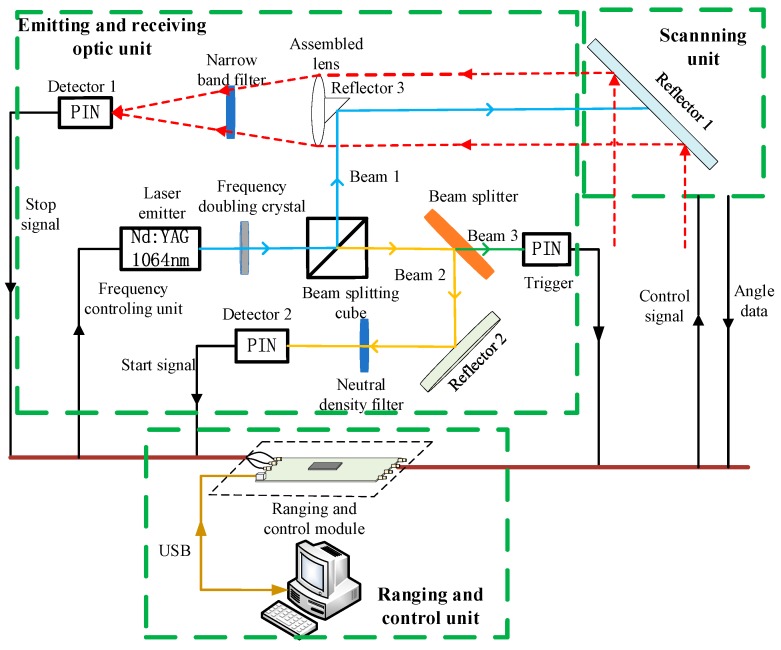
Lab-build LiDAR system.

To acquire the accurate time interval between the emitted and the returned pulse, a pulsed TOF LiDAR system was constructed based on the time-pickoff circuit including AGC and timing discriminators [17]. An assembly view of the lab-build LiDAR system appears in Figure 1. It is divided into three parts: emitting and receiving unit, ranging and control unit and scanning unit.

In the emitting and receiving unit, a solid state Q-switched laser set at 1064 nm is employed as the laser emitter, whose repetition frequency is from 1 kHz to 5 kHz. The energy of a laser pulse is equal approximately to 18.71 μJ. The full width at half-maximum (FWHM) of laser pulse is nominally 10 ns. As shown in Figure 1, the emitted laser pulses are split into three beams by the beam splitting cube and the beam splitter, which are the emitted pulse toward target (beam 1), the emitted pulse as start signal (beam 2) and the trigger pulse (beam 3). The laser beam reflected from the targets is gathered by the assembled lens with a diameter of 35 mm. Then the collected laser beam is converted into an electrical signal by the photoelectric detector 1. This electrical signal is fed to the ranging and controlling unit as stop signal. Beam 2 is detected by the photoelectric detector 2 after reflection and attenuation. The electrical signal converted from beam 2 is fed to the ranging and controlling unit as start signal. Beam 3 is collected as a trigger for three-dimensional point cloud imaging.

The scanning unit is used to detect targets in three-dimensional directions. A vertical scan is completed by a stepper motor and a horizontal scan is completed by a controllable holder. The ranging and control unit is mainly used to complete the functions of two parts, the time interval measurement and the system control. This paper used Field-Programmable Gate Array (FPGA) to configure parameters of the laser emitter, the stepper motor, AGC and Time-to-Digital Converter (TDC), and eventually receive feedback signals from AGC. Besides, FPGA gathers, processes and uploads data to a computer through USB connector as well.

The main function of the ranging and controlling unit is to measure the time interval between start signal and stop signal, which is proportional to the target distance. This paper applies a TDC chip to measure the time interval because of its high integration and high linearity. A TDC-GP22 is used in the proposed LiDAR system, which is of high precision and the resolution of 45 ps for dual channels (the emitting channel and the receiving channel). Furthermore, in order to reduce the walk error and timing jitter appearing in the returned channel (stop signal), this paper proposes a time-pickoff circuit based on AGC and timing discriminators mainly including CFD to ensure the accurate time interval has high accuracy and precision.

### 2.2. Design of AGC

Since the pulse amplitude is inversely proportional to the square of the distance and is proportional to the target reflectance, the returned pulse amplitude changes widely in large dynamic range. Before the input pulse is sent into the timing discriminators, its amplitude is stabilized into a fixed range by an AGC [7].

**Figure 2:**
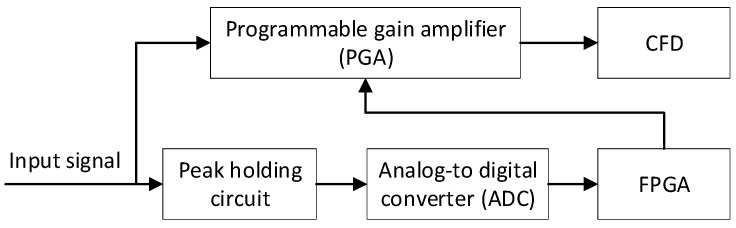
Schematic diagram of AGC.

A schematic diagram of the AGC is shown in Figure 2. The input pulse is divided into two paths. One is transmitted to the peak holding circuit, which is sampled by the Analog-to-Digital Converter (ADC), and then the output value of ADC is stored by FPGA. The other is fed to the Programmable Gain Amplitude (PGA), which is controlled by FPGA according to the peak value of the input pulses. The input pulses are divided into several levels according to their amplitudes based on LiDAR equation.

The peak holding circuit is designed to maintain the pulse peak for a period of time (more than 1 μs) for readily sampling the input pulse by using ADC. OPA615, a holding capacitor (30 pF) and a Schottky diode (BAT17) are used to complete peak holding, which has a great advantage in transconductance peak holding. ADC is utilized to detect and sample the output of the peak holding circuit. In order to satisfy the repetition frequency of the laser (5 kHz), ADS7884 is selected to sample the output of the peak holding circuit which has a sampling rate of 3 MHz. The selection of the PGA depends on the range of the returned pulse amplitude, which need to be calculated as follows. PGA870, which has a dynamic gain in the range of −11.5~20 dB, is selected as the PGA in the AGC system. Here, ADS7884 and PGA870 are able to be programmable controlled by FPGA.

According to the LiDAR equation, the dynamic ranges of peak power of the returned pulse are calculated into a fixed range. Assuming that the detected distance covers from 10 m to 100 m, the peak power of the returned pulse can be written as:

(1) $$P_{r}=P_{T}\eta_{o}\rho \frac{A_{R}}{\Omega_{Ta}R^{2}}\eta_{\alpha }^{2}$$

where $P_{T}$ and $P_{r}$ represent peak power of the emitted and returned pulse (W), respectively, $\eta_{o}$ is transmission factor the optical system, ρ is the reflectance of the target, $A_{R}$ is the receiver area (m^2^), $\Omega_{Ta}$ is the scattering angle of targets, R is the target range (m) and $\eta_{\alpha }^{2}$ represents the two-way atmospheric transmission.

The target is assumed to be Lambertian, thus $\Omega_{Ta}$ is considered as π. Here, ρ is usually varied from 0.2 to 0.8 [18]. $A_{R}$ is equal to a circle area with a diameter of 35 mm in the lab-build LiDAR system. $\eta_{o}$ primarily refers to the transmittance and reflectance of the beam-splitter cubes and the reflecting mirror, which are optical devices of the LiDAR. Therefore, $\eta_{o}$ is calculated as:

(2) $$\eta_{o}=\eta_{\mathrm{ob}}\times \eta_{\mathrm{or}3}\times \eta_{\mathrm{or}1}$$

where $\eta_{\mathrm{ob}}$ is reflectance of the beam-splitter cubes (0.99), $\eta_{\mathrm{or}3}$ and $\eta_{\mathrm{or}1}$ are the reflectance of the reflector 3 and the reflector 1 (0.9, 0.9), as shown in Figure 1. The shape of the emitted pulse satisfies Gaussian distribution in time domain, so the peak power of the emitted pulse is:

(3) $$P_{T}=\frac{E_{T}}{P_{w}}$$

where $E_{T}$ represents energy per emitted pulse (J) and $P_{w}$ represents the width of the received pulse (s). The typical $\eta_{\alpha }^{2}$ is approximately 0.98. Combining Equations (1) and (3), we rewrote Equation (1) in the following form:

(4) $$P_{r}=\eta_{o}\rho \frac{{E_{T}A}_{R}}{P_{w}\pi R^{2}}\eta_{\alpha }^{2}$$

According to Equation (4), if the target range is in the scope from 10 m to 100 m, the corresponding peak power of the returned pulse can be calculated to be from 3.6 × 10^−3^ W to 9.0 × 10^−6^ W (ρ=0.2∼0.8). The amplitude of photodetector output ($A_{P}$) can be written as:

(5) $$A_{P}=R_{\lambda }P_{r}R_{f}$$

where $R_{\lambda }$ is the unity-gain responsivity of the detector (A/W), *R_f_* is the feedback resistance in the detected circuit (kΩ). In this system, $R_{\lambda }=0.6$ A/W and *R_f_* = 10 kΩ. Combining the equations above, the returned pulse amplitude is in the range from 0.05 V to 3 V, which is the input pulse amplitude of the AGC circuit. The output of AGC circuit is fed to the CFD in the timing discriminator, thus the AGC input should be adjusted to satisfy the input scope of the timing discriminator, which should range from 1 V to 2.5 V. The AGC output can be changed through setting the gain of the PGA in the AGC circuit. As presented in Table 1, the PGA gain in the AGC circuit is divided into seven levels to concentrate the input pulse amplitude (0.05–3 V) into a limited range (1–1.5 V). Through optimizing the PGA gain in the AGC circuit, the CFD detection performance can be improved.

### 2.3. Design of Timing Discriminators

**Figure 3:**
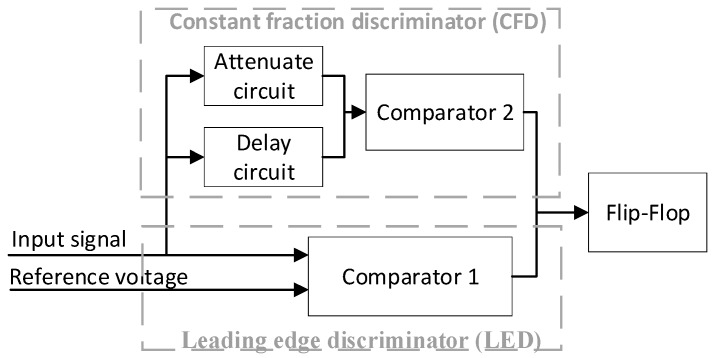
Schematic diagram of the timing discriminators.

A timing discriminator is used to convert the analog electrical pulses into emitter coupled logic (ECL) pulses at the right instant [7]. This paper employs a timing discriminator including CFD and leading edge discriminator (LED), as shown in Figure 3. CFD is designed to reduce for walk error caused by the pulse amplitude and rise time [5]. LED produces a triggering pulse when the input pulse crosses a constant level, which is a feasible way to avoid false triggers resulting from noise.

The AGC output signal is fed to the delay circuit and the attenuation circuit. When the delayed signal crosses the attenuated signal, a zero-crossing point ($t_{T}$) is obtained to produce a logic pulse to trigger TDC. The zero-crossing points in the returned pulse are regarded as the returned moment. The measured time interval is calculated from the difference between the emitted moment and the returned moment. Theoretically speaking, the zero-crossing point ($t_{T}$) is independent from the pulse amplitude.

Two physical parameters, delay time ($t_{d}$) and attenuation fraction (k), are the most significant circuit parameters for the acquisition of the accurate zero-crossing point ($t_{T}$). In several reports, the attenuation fraction (k) was implied to be adjusted in the range from 0.2 to 0.5 [19], otherwise the range precision may deteriorate significantly. In this paper, the determination of the attenuation fraction and the delay time in CFD circuit were discussed in depth to ensure a high range precision.

## 3. Methodology

The Cramer−Rao Lower Bound (CRLB) provides a lower limit on the variance of any unbiased estimate of a parameter such as range [20]. CRLB on range estimate has been derived in previous literature [9]. Here, it was described briefly for the calculation for CRLB on range estimates in Section 3.1. In Section 3.2, the timing jitter as the main random error of the time-pickoff circuit is calculated based on a certain amount of electronic noise. Through the analysis of CRLB and the estimation of the timing jitter, it is obvious that the range precision is closely related to several key parameters, such as pulse amplitude (A), pulse width ($p_{w}$), delay time ($t_{d}$) and attenuation fraction (k). In Section 3.3, the noise analysis can provide a regular basis for Monte Carlo simulation of range estimation which is elaborated in Section 3.4. Furthermore, the simulation analysis can provide a way of discussing the relationship between the circuit parameters and pulse parameters and range precision of LiDAR in the next section.

### 3.1. Cramer–Rao Lower Bound on Range Estimate (CRLB)

CRLB can be used to analyze the range precision, since it provides a lower limit for the variance of range estimates or any other unbiased estimate. The typical laser pulse is modeled as a Gaussian shape, heavy-tailed or truncated parabola, which have been employed in previous researches [9]. Based on the shape of the laser pulse in the lab-build LiDAR, the truncated parabola model is suited to express the temporal shape of the returned pulse as:

(6) $$I(t_{k})=A[1-\left(\frac{t_{k}-2R/c}{p_{w}}\right){}^{2}]rect(\frac{t_{k}-2R/c}{2p_{w}})+B$$

where, $I(t_{k})$ represents the number of photons that are detected in one sample, A refers to the pulse peak amplitude (V), R is the detected range (m), c is the speed of light (m/s), $p_{w}$ represents the pulse width (s) (the total pulse width is $2p_{w}$, the FWHM is $\sqrt{2}p_{w}$). B is the bias level of the signal (V), $t_{k}$ is the time when the *k*th sample is collected (s). The function denoted rect() is defined as a rectangle function. For further description of the calculation of CRLB readers may refer to the literature [9,20].

Since the photon arrival during a sampling interval is assumed as a Poisson process, the noise in LiDAR system is in Poisson distribution. The Poisson probability density function (PDF) can be described as [9]:

(7) $$P_{D(t_{k})}=\mathrm{Pr}ob[D(t_{k})=d(t_{k})]=\frac{1}{d(t_{k})!}{I_{\left(t_{k}\right)}}^{d(t_{k})}\exp[-I(t_{k})]$$

where, $D(t_{k})$ is a Poisson distributed random variable, which refers to the photon arriving at $t_{k}$, $d(t_{k})$ is the photocount realizations, the function $\mathrm{Pr}ob[D(t_{k})=d(t_{k})]$ represents the probability of the photocount realizations at time $t_{k}$. Since photon arrival is a Poisson process, $d(t_{k})$ is independent of each other for the random arrival of the photons [9]. Thus, the progress of total K samples can be described by the log-likelihood function:

(8) $$l(R)=-\sum_{k=1}^{K}\log[d(t_{k})!]+\sum_{k=1}^{K}d(t_{k})\log[I(t_{k})]-\sum_{k=1}^{K}I(t_{k})$$

where, l(R) is the log-likelihood function associated with the PDF from Equation (7). According to the log-likelihood function of R, the element of the Fisher Information Matrix (FIM) *J_RR_*, which is associated with R, can be deduced as [9,20]:

(9) $$J_{RR}=-E[\frac{\partial^{2}l(R)}{\partial R^{2}}]\approx \frac{32Af_{sam}}{c^{2}p_{w}}[\sqrt{\frac{A+B}{A}}\text{arctan}h(\sqrt{\frac{A+B}{A}})-1]$$

where $f_{sam}$ represents the sampling frequency. CRLB can be obtained by inverting the FIM *J_RR_*, and the CRLB on the range estimate is the inverse of the FIM element associated with the range [12]. Therefore, the CRLB aiming at a signal mixed with Poisson noise can be rewritten as:

(10) $$\mathrm{var}[\overset{⌢}{R}]\geq \frac{c^{2}p_{w}}{32Af_{sam}[\sqrt{\frac{A+B}{A}}\text{arctan}h(\sqrt{\frac{A+B}{A}})-1]}$$

where the function $\mathrm{var}[\overset{⌢}{R}]$ represents the minimum variance of the unbiased range estimate of the parabolic pulse with Poisson noise. The CRLB derived by assuming Gaussian noise of equal variance can be derived as the same way of Poisson noise, which is [9]:

(11) $$\mathrm{var}[{\overset{⌢}{R}}_{g}]\geq \frac{3Bc^{2}p_{w}}{32A^{2}f_{sam}}$$

Comparing Equation (10) with Equation (11), the CRLB derived by assuming Poisson noise is always greater than the CRLB derived by assuming Gaussian noise. In this paper, the CRLB of the range estimate is derived by Gaussian noise.

### 3.2. Timing Jitter 

**Figure 4:**
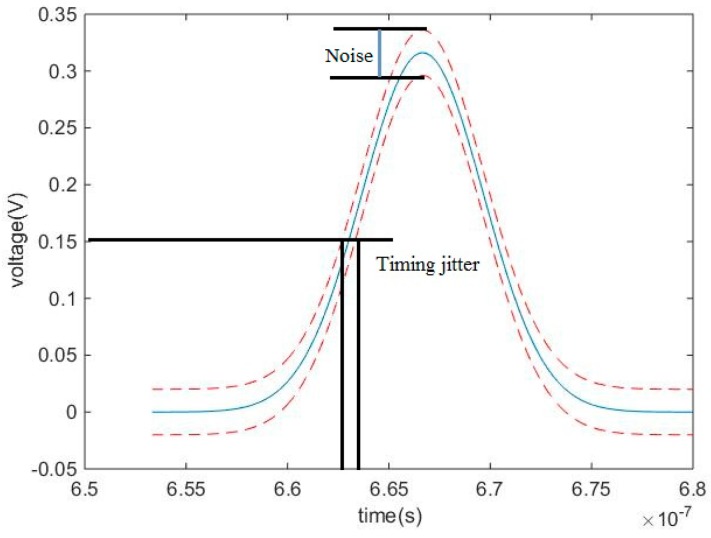
Definition of TJ caused by noise as statistical fluctuations existing in the shape of the detected pulses.

Timing jitter (TJ) is defined as the timing error caused by noise such as statistical fluctuations existing in the shape of the detected pulses, as shown in Figure 4. The precision of the LiDAR system is mainly determined by timing jitter [5]. Timing jitter can be used to indicate the high limit of the standard deviation (StD) of the range measurement data. Timing jitter is calculated by the ratio of the root mean square (RMS) of noise to the slope of the returned pulse at the timing moment [5,6].

Noise following the returned signal enters into attenuation circuit and delay circuit of CFD. The part of noise entering into the attenuation circuit of CFD is attenuated *k* times. The remaining part of the noise entering into the delay circuit is not changed. The RMS of noise is assumed as $V_{no}$, the output noise of comparator $f_{no}$ can be written as:

(12) $$f_{no}=V_{no}\sqrt{1+k^{2}}$$

where, *k* is the attenuation fraction. As described in Section 3.1, the laser emitted pulse is modeled as truncated parabola. The tiny bias level B is ignored, so that we can obtain the delayed signal ($f_{d}(t)$) and the attenuated signal ($f_{a}(t)$) as:

(13) $$f_{d}(t)=A(1-\frac{{\left(t-t_{d}\right)}^{2}}{{p_{w}}^{2}})rect(\frac{t-t_{d}}{2p_{w}})$$

(14) $$f_{a}(t)=kA(1-\frac{t^{2}}{{p_{w}}^{2}})rect(\frac{t}{2p_{w}})$$

The zero-crossing point $t_{T}$ is the moment when the delayed signal crosses the attenuated signal. The pulse amplitude in the time of $t_{T}$ satisfies:

(15) $$A(1-\frac{{\left(t_{T}-t_{d}\right)}^{2}}{{p_{w}}^{2}})=kA(1-\frac{{t_{T}}^{2}}{{p_{w}}^{2}})$$

where $t_{d}$ refers as the delay time. According to Equation (15), $t_{T}$ can be calculated as:

(16) $$t_{T}=\frac{t_{d}-\sqrt{{{kt}_{d}}^{2}+{\left(1-k\right)}^{2}{p_{w}}^{2}}}{1-k}$$

Combining Equations (13), (14) and (16), the voltage difference between the input terminals of the comparator can be calculated as:

(17) $$f_{c}(t)=-\frac{A(1-k)}{{p_{w}}^{2}}t^{2}+\frac{2At_{d}}{{p_{w}}^{2}}t+A(1-k)-\frac{{{At}_{d}}^{2}}{{p_{w}}^{2}}$$

Based on the definition, timing jitter $\sigma_{t}$ can be written as:

(18) $$\sigma_{t}=\left|\frac{f_{no}(t)}{{\frac{df_{c}(t)}{dt}|}_{t_{T}}}\right|=\frac{V_{no}\sqrt{1+k^{2}}{p_{w}}^{2}}{2A\sqrt{{{kt}_{d}}^{2}+{\left(1-k\right)}^{2}{p_{w}}^{2}}}$$

The ranging deviation resulted from the timing jitter can be calculated as:

(19) $$\sigma_{l}=\frac{{c\sigma }_{t}}{2}=\frac{{cV}_{no}\sqrt{1+k^{2}}{p_{w}}^{2}}{4A\sqrt{{{kt}_{d}}^{2}+{\left(1-k\right)}^{2}{p_{w}}^{2}})}$$

Equation (19) shows that the timing jitter is related with the pulse amplitude (A), pulse width ($p_{w}$), the attenuation fraction (k) and the delay time ($t_{d}$).

### 3.3. Sources of Noise in a LiDAR System

In order to simulate the noise applied in Monte-Carlo simulation, the noise sources of a LiDAR system, which consist of background noise, photo-diode noise and pre-amplifier circuit noise [21], are discussed in this section,.

#### 3.3.1. Background Noise

The sources of the background noise are mainly divided into three parts: thermal radiation from self-emitting sources, solar radiation reflected by the background and radiation scattered by the atmosphere [22]. The solar radiation is far greater than the other two kinds of radiation, so that the background noise is mainly determined by the solar radiation. According to the previous researches [21], the noise current caused by the solar radiation can be written as:

(20) $$I_{B}=R_{\lambda }N_{\lambda }\Omega_{FOV}A_{R}\eta_{\alpha }\eta_{o}\Delta \lambda$$

where $N_{\lambda }$ is the spectral radiance of a background source (W·m^−2^·sr^−1^·μm^−1^), $\Omega_{FOV}$ is the solid angles of receiving Field Of View (FOV), Δλ is the FWHM of the neutral-density filter (μm). As described in Section 2.2, the unity-gain responsivity of the detector $R_{\lambda }$ (A/W), the atmosphere transmittance at 1064 nm $\eta_{\alpha }$ and the transmission factor of the optical system $\eta_{o}$ are 0.6, 0.98 and 0.8, respectively.

Assuming that the solar radiation is reflected by the target, $N_{\lambda }$ can be calculated by $E_{s}\rho_{B}$, where $E_{s}$ represents the solar irradiance per steradian (W/m^2^·sr·μm). $E_{s}$ is approximately 500 W/m^2^·μm for perpendicular incident sunlight [22]. The reflectance $\rho_{B}$ of the background is assumed as 0.4, since the LiDAR system is often used in the city and the material of the background is concrete. According to the datasheet of the applied PIN photodiodes [23], the bandwidth of the neutral density filter Δλ is 10 × 10^−3^ μm. $\Omega_{FOV}$ is described as D/2f, where f is the focal length of objective lens (71 mm) and D is the diameter of the telescope equalling 35 mm. Thus, the noise current $I_{B}$ is equal to 2.231 × 10^−4^ A.

**Figure 5:**
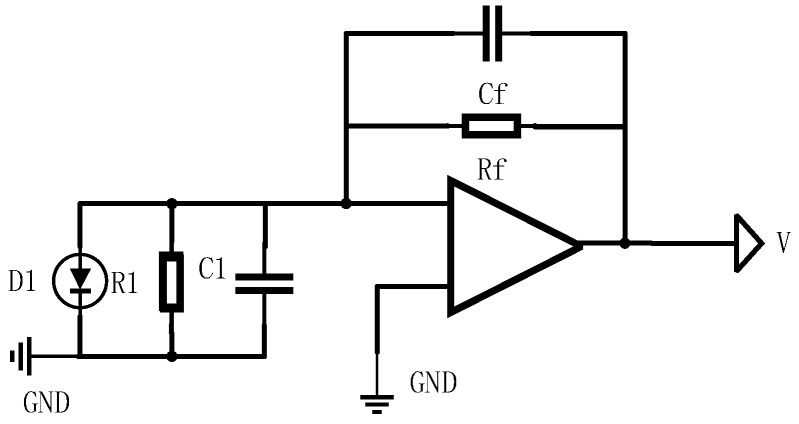
The schematic diagram of pre-amplifier circuit.

The RMS of the background noise current is calculated as [21]:

(21) $${\bar{i}}_{B}=\sqrt{\left(2qI_{B}B_{w}\right)}$$

where, q represents the charge of electron, which is equal to 1.6 × 10^−19^ C. The signal bandwidth of the pre-amplifier circuit $B_{w}$ (Hz) can be calculated by:

(22) $$B_{w}=\frac{1}{2\pi R_{f}C_{f}}$$

where *R_f_* and *C_f_* are the feedback resistance and capacitor in the pre-amplifier circuit. The schematic diagram of pre-amplifier circuit is shown in Figure 5, whose parameters are set as follows: *R_f_* = 10 kΩ, *C_f_* = 0.5 pF, $C_{1}=5$ pF, $R_{1}=1000$ MΩ. According to the above parameters, the RMS of the background noise current can be rewritten as ${\bar{i}}_{B}=4.767\times 10^{-8}$ A.

According to Equations (21) and (22), the RMS voltage of the background noise ${\bar{u}}_{B}$ (V) can be calculated by:

(23) $${\bar{u}}_{B}={\bar{i}}_{B}R_{f}=4.767\times 10^{-4}$$

#### 3.3.2. Photodiode Noise

The noise caused in photodiodes by dark current and shot noise is usually described by noise equivalent power (NEP) in the datasheet of PIN photodiodes. The RMS of the equivalent current of photodiode noise (A) can be written as:

(24) $${\bar{i}}_{P}=R_{\lambda }D_{NEP}\sqrt{B_{w}}$$

where $D_{NEP}$ represents the noise equivalent power ($W/\sqrt{\mathrm{Hz}}$). Based on the datasheet of the PIN photodiode, the typical value of $D_{NEP}$ equals $4.2\times 10^{-15}W/\sqrt{\mathrm{Hz}}$ [23]. The RMS voltage of photodiode noise ${\bar{u}}_{P}$ (V) is calculated by:

(25) $${\bar{u}}_{P}={\bar{i}}_{P}R_{f}=1.422\times 10^{-7}$$

#### 3.3.3. Pre-Amplifier Circuit Noise

The pre-amplifier circuit noise consists of the voltage noise and the current noise caused by the operational amplifier (OPAMP), and the thermal noise caused by resistance [24]. Each kind of noise is discussed as follows: firstly, the input voltage noise and the input current noise can be converted to the output RMS voltage as [24]:

(26) $${\bar{u}}_{V}=NG\times \sqrt{1.57f_{c}\times u_{vi}^{2}}$$

(27) $${\bar{u}}_{I}=NG\times \sqrt{1.57f_{c}\times R_{f}\times u_{ii}^{2}}$$

where ${\bar{u}}_{V}$, ${\bar{u}}_{I}$ are the output voltage noise and current noise (V), NG represents the noise gain of the pre-amplifier circuit, $f_{c}$ is the closed loop bandwidth in the pre-amplifier circuit (Hz). $u_{vi}$ ($V/\sqrt{\mathrm{Hz}}$) and $u_{ii}$ ($A/\sqrt{\mathrm{Hz}}$), the input voltage noise and current noise of the OPA657, are equal to $4.8\times 10^{-9}\;V/\sqrt{\mathrm{Hz}}$ and $1.3\times 10^{-15}A/\sqrt{\mathrm{Hz}}$, which are given as a constant in the datasheet [25]. The value of $f_{c}$ in the pre-amplifier circuit is equal to the signal bandwidth $f_{s}$, which is illustrated in Equation (28) [24]. The noise gain *NG* can be calculated by Equation (29):

(28) $$f_{c}=f_{s}=1/(2\pi R_{f}C_{f})$$

(29) $$NG=1+C_{1}/C_{f}$$

where $C_{1}$ and $C_{f}$ is the parameters in the pre-amplifier circuit, which is shown in Figure 5. Combining Equations (26)–(29), the output RMS voltage of the voltage noise and the current noise can be written as ${\bar{u}}_{V}=3.733\times 10^{-4}$ V, ${\bar{u}}_{I}=1.011\times 10^{-8}$ V, respectively.

Secondly, as shown in Figure 5, the thermal noise caused by resistor *R_f_* and $R_{1}$, ${\bar{u}}_{T1}$ are ${\bar{u}}_{T2}$ (V), described as:

(30) $${\bar{u}}_{T1}=\sqrt{4k_{B}TR_{1}\times 1.57f_{s}}$$

(31) $${\bar{u}}_{T2}=\sqrt{4k_{B}TR_{f}\times 1.57f_{s}}\times R_{f}/R_{1}$$

where, $f_{s}$ is the signal bandwidth, $k_{B}$ is Boltzmann constant, which is equal to $1.3806505\times 10^{-23}$ J/K. T represents a temperature in Kelvins, which is assumed as 298.15 K [24]. The total RMS voltage of thermal noise (V) in the pre-amplifier circuit can be calculated by:

(32) $$u_{T}=\sqrt{{{\bar{u}}_{T1}}^{2}+{{\bar{u}}_{T2}}^{2}}=9.071\times 10^{-5}$$

In summary, the RMS voltage of the total output noise is obtained by combining the above individual parts in a root-sum-squares manner, so the RMS voltage of noise (V) in the first operational amplifier of the pre-amplifier circuit is calculated by:

(33) $$\bar{u}=\sqrt{{{\bar{u}}_{B}}^{2}+{{\bar{u}}_{P}}^{2}+{{\bar{u}}_{V}}^{2}+{{\bar{u}}_{I}}^{2}+{{\bar{u}}_{T}}^{2}}=6.122\times 10^{-4}$$

Since another operational amplifier is employed in the pre-amplifier circuit and that the output signal of the pre-amplifier is send to the time-pickoff circuit, the noise following the signal would be amplified by 15 times, which is the product of the gain of the second operational amplifier and the gain of the time-pickoff circuit. Thus, the RMS voltage of the noise in the time-pickoff circuit is approximately ${\bar{u}}_{t}=9.18\times 10^{-3}$ V. This estimation value will be used for Monte Carlo simulation of range estimation in the next section.

### 3.4. Range Standard Deviation in Monte Carlo Simulation

**Figure 6:**
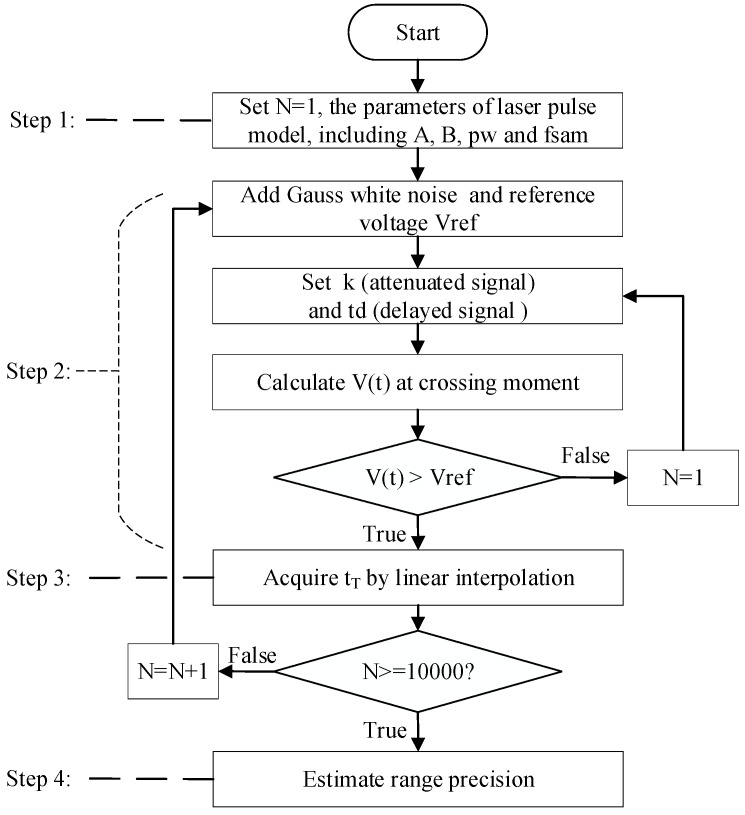
Flow diagram of Monte Carlo simulation for estimating the range precision.

Monte Carlo simulation is a method that uses multiple estimates to achieve all possible solutions, according to the law of large numbers. Thus, it can be used to estimate the performance range of the LiDAR system through analyzing statistically the distribution of the simulated results of range estimate. In this paper, the Monte Carlo simulations employ CFD to estimate the time of the returned pulse, which is proportional to range. The statistical simulated results can be illustrated the influence of pulse amplitude (A), pulse width ($p_{w}$), attenuation fraction (k) and delay time ($t_{d}$) on the range precision. The flow chart of the Monte Carlo simulation appears in Figure 6. For the effectiveness of Monte Carlo simulation, each simulation is carried out 10,000 times, so as to obtain enough estimate data for the requirements of the Monte Carlo simulation.

At step 1, the emitted laser pulse is modeled as a truncated parabola, whose parameters include the pulse amplitude (*A*), pulse width ($p_{w}$), and bias level (B), according to Equation (6). At step 2, the added noise is assumed as white Gaussian noise, whose RMS can be calculated using Equation (33) in Section 3.3. The reference voltage $V_{ref}$ is used to avoid false triggers caused by the noise, whose value is 0.07 V. The sampling frequency is set as 100 GHz. The noisy signal is divided into two paths, one of which is attenuated by k times and the other is delayed by time $t_{d}$. If the delayed signal crosses the attenuated signal, the signal amplitude at the moment of crossing are denoted as *V*(*t*). According to the operation principle of the time discriminators, the zero-crossing point $t_{T}$ can be acquired only when *V*(*f*) is greater than the reference voltage *V_ref_* of LED. In the case that *V_ref_* did not reach the amplitude threshold, we need to reset the circuit parameters including the attenuation fraction k and the delay time $t_{d}$. If $V(t)\geq V_{ref}$, a rough calculation of the zero-crossing point can be obtained. In order to calculate the zero-crossing point accurately, linear interpolation is implemented for obtaining the accurate $t_{T}$ at step 3. The average and StD of the ranging data would be estimated at step 4, which can illustrate the range precision.

## 4. Simulation Results and Discussion

### 4.1. Simulation Results for Pulse Amplitude (A)

**Figure 7:**
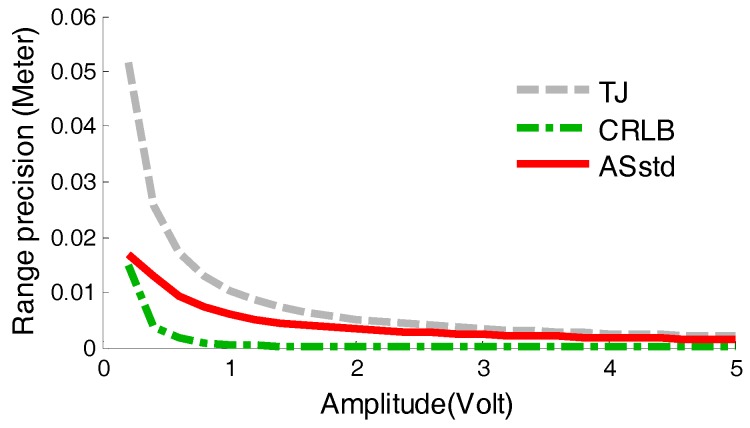
Comparison of ASstd, CRLB and TJ. TJ means the time jitter calculated from Equation (19), CRLB means the Cramer–Rao Lower Bound on range estimated from Equation (11), and ASstd values are the measured range StD calculated based on Section 3.4.

In the simulations, the parameters were set as follows: *p_w_* = 7 ns, *B* = 10 mV, *k* = 0.5, *t_d_* = 2 ns, *f_sam_* = 100 GHz. The reference voltage *V_ref_* of LED in the timing discriminators was 0.07 V. The simulations were established for 25 groups according to the pulse amplitude A, which was varied from 0.2 V to 5 V with a change step of 0.2 V. At each amplitude, 10,000 pulse amplitude simulation results were collected and thus the amplitude simulation based StD were calculated, which was called ASstd for short, and indicated by the red solid line shown in Figure 7. Besides, CRLB and TJ values varied with the amplitude are estimated according to Equations (11) and (19), as shown in Figure 7.

Based on the results shown above, two conclusions can be reached as follows: firstly, the ASstd value is between TJ and CRLB, because CRLB is the low limit of the range estimation deviation and TJ refers to the maximum range estimation deviation. Secondly, the amplitude-based StD of the three kinds of estimation decrease with the increase of the amplitude, which obeys the physical law.

### 4.2. Simulations Results for Pulse Width (pw)

**Figure 8:**
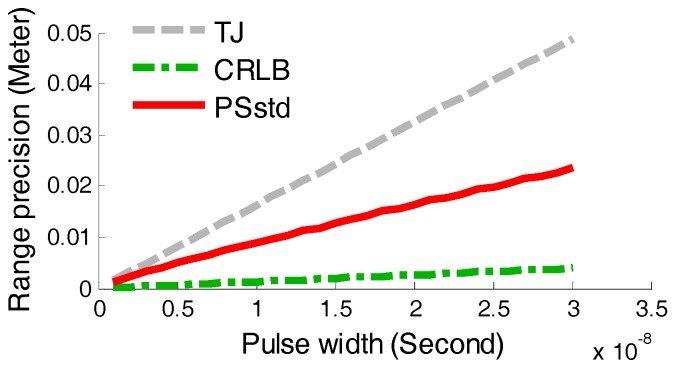
Comparison of PSstd, CRLB and TJ.

In this section, the pulse width $p_{w}$ is adjusted in the range from 1 ns to 30 ns with a change step of 1 ns. In order to ensure that the zero-crossing point $t_{T}$ lies along the rising edge of the attenuated signal, the delay time $t_{d}$ need to be varied with the pulse width. Previous researches have indicated that the relationship between the delay time and the rise time $t_{r}$ of the returned pulse is $t_{d}=(1-k)t_{r}$ [26]. The rise time of the returned pulse $t_{r}$ is proportional to the pulse width $p_{w}$, so that here we set the delay time as $t_{d}=(1-k)p_{w}$. The pulse amplitude was set as A=0.8 V and other parameters were the same as in Section 4.1. The pulse width simulation-based StD (PSstd) values were compared with CRLB and TJ, as described in Figure 8.

As seen from Figure 8, it can be concluded that the range precision will deteriorate with the increase of the pulse width, which can be deduced from Equations (11) and (19). Thus, if the pulse width was expanded because of the larger slope and roughness of the target or light spot expansion in long distance, the range precision would be reduced along with the augmented pulse width. Because the emitted pulse width has been determined to be one fixed value according to the performance of the time-pickoff circuit, the pulse width based experiments were not performed.

### 4.3. Simulation Results for Attenuation Fraction (k) and Delay Time (td)

**Figure 9:**
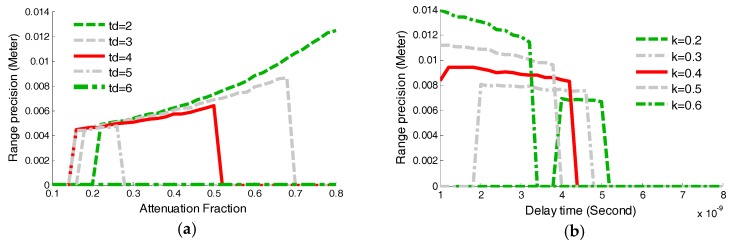
The variations of KSstd and DSstd as the increase of the attenuation fraction and the delay time. (**a**) The variation of KSstd with different $t_{d}$; (**b**) The variation of DSstd with different k.

In order to design and optimize the time-pickoff circuit, this section mainly focused on the attenuation fraction simulation-based StD (KSstd) and delay time simulation-based StD (DSstd), which can indicate how the range precision is influenced by the attenuation fraction (k) and delay time ($t_{d}$). As shown in Figure 9a, the attenuation fraction k was changed from 0.1 to 0.8 with a change step of 0.02. Five groups of simulations for different delay times $t_{d}$ were established, which was varied from 2 ns to 6 ns in increments of 1 ns. As shown in Figure 9b, five groups of simulations were performed with different attenuation fractions k ranging from 0.2 to 0.6. The delay time $t_{d}$ was changed from 1 ns to 8 ns incremented in steps of 0.02. In all simulations, the pulse amplitude was set to be A=0.8 V and the pulse width was 7 ns.

Based on these results shown in Figure 9a,b, three conclusions can be reach, summarized as follows: firstly, k should be set in the range from 0.2 to 0.6 which can guarantee high precision LiDAR ranging. As red solid-line shown in Figure 9a illustrates, an attenuation fraction k in the range from 0.2 to 0.6 is essential for range measurement. If the minimal k is less than 0.2, the $t_{d}$ employed in the time-pickoff circuit could push the delayed signal to miss the attenuated signal and the zero-crossing point cannot be obtained. Secondly, the range precision decreases as the value of *t_d_* is increased. However, there is an upper limit on the delay time $t_{d}$ when the attenuation fraction k is fixed, as shown in the Figure 9b. Thirdly, when the attenuation fraction k is determined, the delay time $t_{d}$ can be adjusted within a selected range. As shown in Figure 9b, the width of the selected-range of k=0.4 (red solid line) is the largest; on the contrary, the width of the selected-range of k=0.2 (green dashed line) is the smallest.

### 4.4. Influence of Pulse Amplitude on DSstd

**Figure 10:**
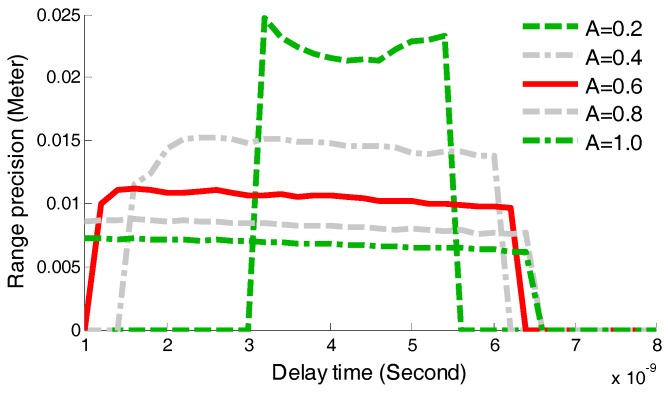
Comparison of DSstd varied with the different amplitudes.

In order to discuss how the DSstd values are influenced by the pulse amplitude A, five groups of simulations were established through adjusting the pulse amplitudes with a change step of 0.2 V from 0.2 V to 1.0 V in this section. The attenuation fraction k was set as 0.4, and the delay time was changed with a change step of 0.02 ns from 1 ns to 8 ns in every simulation group. The variation of DSstd under different pulse amplitude conditions is illustrated in Figure 10. It is obvious that under a given attenuation fraction, the width of the selected-range of $t_{d}$ decreases with the decrease of the pulse amplitude. If a time-pickoff circuit utilizes $t_{d}$ equaling 2 ns, the range measurement is invalid when the returned pulse amplitude is less than 0.2 V, as green dash-line shown in Figure 10.

## 5. Experiment and Results

In order to verify the relationship between the range precision and the key parameters in the pulse and time-pickoff circuit, main three key parameters are adjusted to verify the range precision, including the pulse amplitude (A), attenuation fraction (k) and delay time ($t_{d}$).

**Figure 11:**
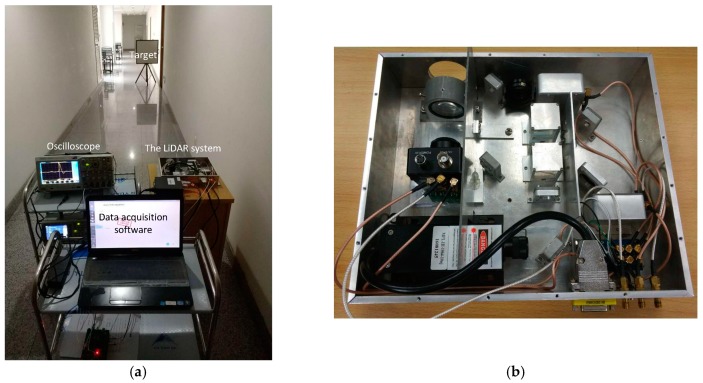
Experiment scene of LiDAR ranging and waveform recording. (**a**) The acquisition software and oscilloscope are used to gather the data. (**b**) The enlarged LiDAR system.

The pulse width $p_{w}$ is nominally 7 ns. In this section, the StD of experiment results for pulse amplitude A (attenuation fraction k and delay time $t_{d}$) is called AEstd (KEstd, DEstd) for short. The definitions of ASstd, KSstd and DSstd used in experiments are same as those in the simulations. The experimental scene is displayed in Figure 11, where the acquisition software and oscilloscope are used to gather the measurement data.The oscilloscope can get the sampled data of the attenuated signal and delayed signal in time-pickoff circuit. The LiDAR system is utilized to measure the range from the target and the sensor. The reference value of the range measurement was detected by BH-AD41, a laser range finder with a high resolution of 0.1 millimeter. The BH-AD41 employs a laser pulse at 650 nm, whose divergence angle is 0.6 m rad. The nominal precision of the range finder is 2 mm, but it only can detect cooperative targets when the target distance is beyond 100 m.

### 5.1. Experiment Results for Pulse Amplitude (A)

**Figure 12:**
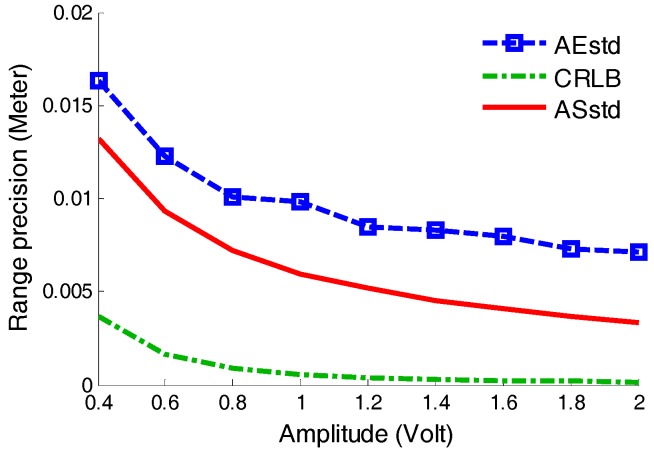
Range precision comparison results for pulse amplitude. AEstd is the amplitude-based experimental StD and ASstd is the amplitude-based simulation StD. CRLB means the Cramer–Rao Lower Bound on range estimates from Equation (19).

In order to study how the pulse amplitude effects the range precision, the attenuation fraction (k) and the delay time ($t_{d}$) in the time pick-off circuit were at fixed values of 0.4 and 4 ns, respectively. Standard diffuse reflectors with reflectivities of 20%, 40%, 60% and 70% were used as targets. Through changing the detected range and the reflectors, nine groups of experiments data can be gathered from the different returned pulse amplitudes. The returned pulse amplitudes were changed from 0.2 V to 2 V with an increase step of 0.2 V. In each group of experiments, 100,000 datapoints were utilized for calculating the range accuracy and precision. The experimental results were compared with the simulation results and CRLB under the same conditions, as shown in Figure 12.

From Figure 12, two conclusions can be obtained as follows. Firstly, AEstd values decrease with the increase of the pulse amplitude *A*. They are greater than the values of ASstd, mostly because the actual noise brought more error into the range measurement. Secondly, the same as the CRLB estimation, AEstd values have a low limiting effect on the range precision when the pulse amplitude is greater than 1.2 V.

### 5.2. Experiment Results for Attenuation Fraction (k) and Delay Time (td)

**Figure 13:**
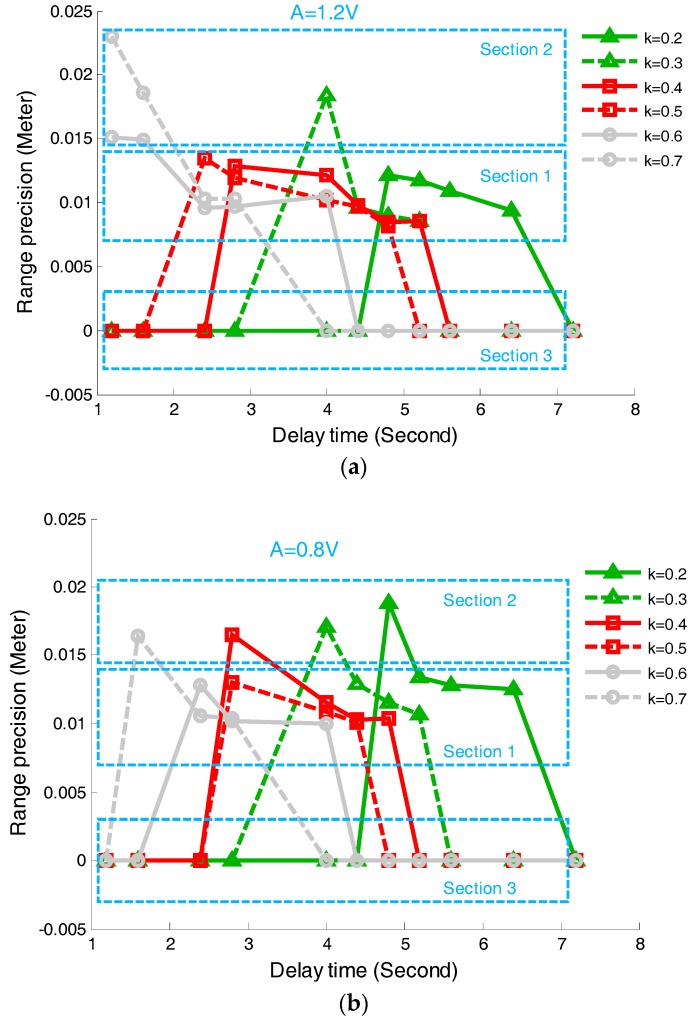
Variation of DEstd with the increase of $t_{d}$ for different pulse amplitudes. (**a**) Relationship between DEstd and $t_{d}$ when A=1.2; (**b**) Relationship between DEstd and $t_{d}$ when A=0.8. DEstd is the delay time-based experimental StD.

A series of experiments were established to verify the influence on the range precision on k and $t_{d}$ in the time pick-off circuit. In the experiments, the returned pulse amplitudes were assigned to be in one of three groups of 0.4 V, 0.8 V and 1.2 V, respectively. Every group has six subgroups according to the different attenuation fractions k, which were varied from 0.2 to 0.7 by changing the resistance value of the attenuation circuit. In each subgroup, $t_{d}$ was adjusted to be 1.2, 1.6, 2.4, 2.8, 4.0, 4.4, 4.8, 5.2, 5.6, 6.4, and 7.2 (ns), respectively, mainly through changing the length of the delayed line in the time pick-off circuit. The experimental results are plotted in Figure 13, where Figure 13a,b illustrate the variance of DEstd varied with the increase of $t_{d}$ under the condition of A=1.2 and A=0.8, respectively.

In Figure 13, DEstd values were divided into three sections: the correct solutions section (Section 1), the terrible solutions section (Section 2), and the no solutions section (Section 3). Based on the results, the following three conclusions can be draw: firstly, $t_{d}$ in the time-pickoff circuit should be determined within the range selected in Section 1, such as 2.80 ns~5.20 ns for k=0.4 (red square solid line), and the range precisions for the selected delay time in this scope are almost the same. Secondly, k in the time-pickoff circuit should be selected depending on the width of the range selected in Section 1. This is because the width of the selected-range reduces with the decrease of the returned pulse amplitudes (*A*). Generally speaking, the parameter values of k are selected within 0.4–0.5 for the best results. Thirdly, compared with the simulation results as shown in Figure 9b, the beginning and ending locations of the range selected in Section 1 as shown in Figure 13 are falling behind the locations of the range selected in Figure 9. This is because a higher reference voltage (150 mV) in the LED was set up to avoid random noise in the experiments, so that the correct zero-crossing point $t_{T}$ is generated by using the larger $t_{d}$. Besides, the actual pulse width would be expanded by the delay lines up to be 9 ns, so that the selected-range of $t_{d}$ is located at a larger $t_{d}$ than in the simulation results.

### 5.3. Selection Criterion of Attenuation Fraction k and Delay Time t_d_

**Figure 14:**
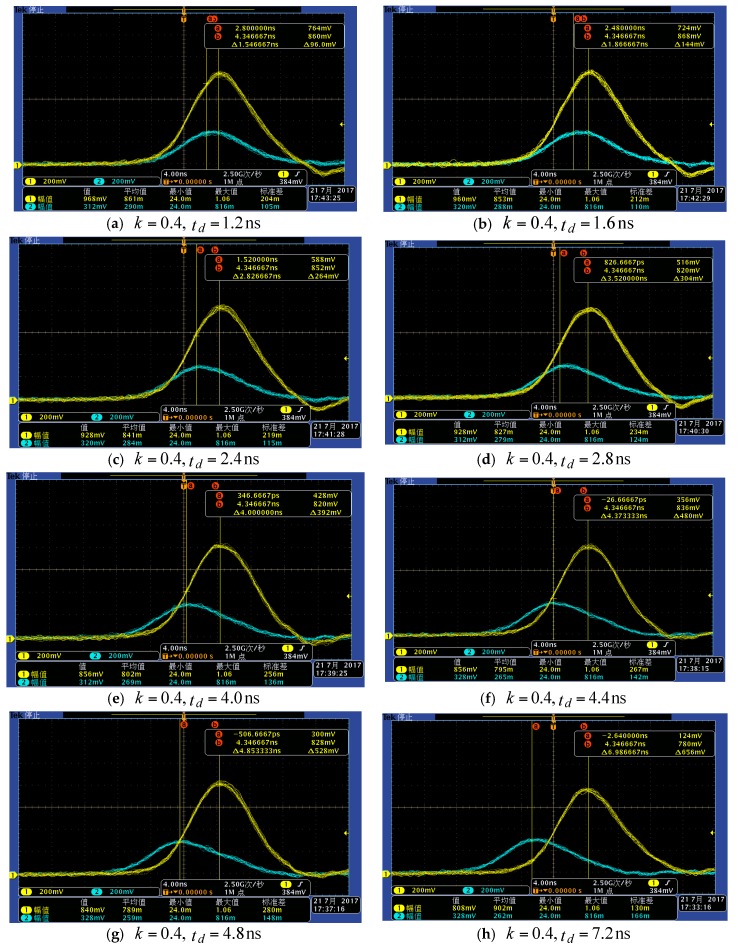
Sampled figures of the attenuated signal and delayed signal. Curve in yellow color represents the delayed signal and curve in cyan color represents the attenuated signal. The delayed signals were moved through adjusting the delay time for obtaining the various the moment of $t_{T}$. All figures are sampled in the case of k=0.4. (**a**) the sampled figure when $t_{d}=1.2$; (**b**) the sampled figure when $t_{d}=1.6$; (**c**) the sampled figure when $t_{d}=2.4$; (**d**) the sampled figure when $t_{d}=2.8$; (**e**) the sampled figure when $t_{d}=4.0$; (**f**) the sampled figure when $t_{d}=4.4$; (**g**) the sampled figure when $t_{d}=4.8$; (**h**) the sampled figure when $t_{d}=7.2$.

**Figure 15:**
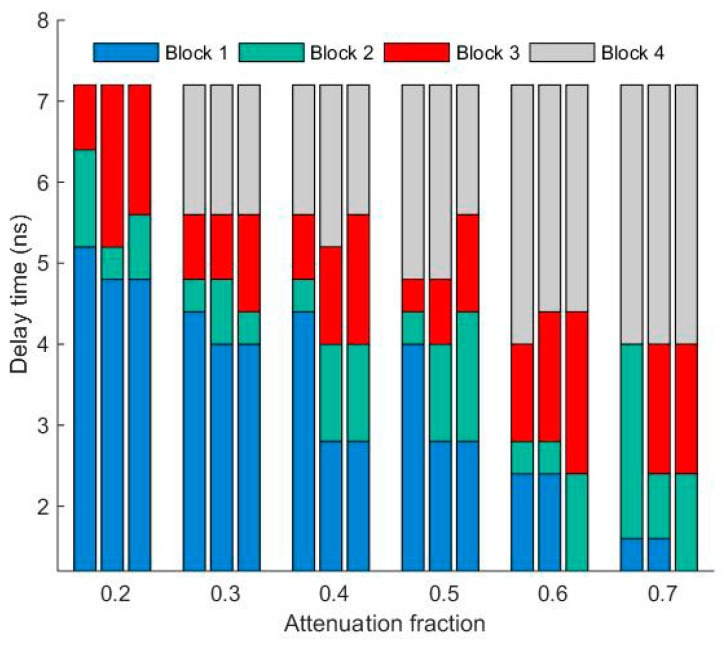
The statistical chart of all data with different $t_{d}$, k and A. Block 1 represents the area of no solution, Block 2 represents the area of terrible solution, Block 3 represents the area of usable solution at the rise edge, Block 4 represents the area of solution at the trailing edge. In one group of experiments, the returned pulse amplitudes were assigned to be 0.4 V, 0.8 V and 1.2 V, and the left one of each three bars is the minimum pulse amplitude (0.4 V). The choice criterion of circuit parameters should follow the range performance (red area) of the minimum pulse amplitude.

In order to discuss intuitively the range precision influence of k and $t_{d}$, the attenuated signal and the delayed signal in the time-pickoff circuit were sampled by an oscilloscope with a 2.5 G sampling rate. For example, the returned pulse under the condition of A=0.8 and k=0.4 are shown in Figure 14, where there are four kinds of situations for the zero-crossing point $t_{T}$, including $t_{T}$ nonexistence (Figure 14a,b), $t_{T}$ existing in noise (Figure 14c), $t_{T}$ on the rise edge (Figure 14d–f), and $t_{T}$ on trailing edge (Figure 14g,h). Three groups of data (amplitude equal to 0.4 V, 0.8 V and 1.2 V) for different attenuation fractions k and delay times $t_{d}$ were obtained, as shown in Figure 14. Therefore, four kinds of results will be discussed by combining Figure 14 and Figure 15.

In Figure 15, Block 1 represents the area of no solution, where the zero-crossing point $t_{T}$ cannot be obtained when the delayed signal and the attenuated signal have no crossing point, as shown in Figure 14a,b. Block 2 represents the terrible solution area. This is because the moment of $t_{T}$ was fluctuating up and down the reference voltage level with noise so that the range precision is beyond the selected-range, as shown in Figure 14c. Block 3 represents the area of usable solutions. The moment of $t_{T}$ occurs at the rise edge of the attenuated signal and exceeds the level of $V_{ref}$, as shown in Figure 14d,f. Block 4 represents the area of solution on the trailing edge. Here $t_{T}$ is obtained at the trailing edge of the attenuated signal, as shown in Figure 14g,h. According to the definition of CFD, the $t_{d}$ values of Block 1 and Block 4 cannot be selected to be employed in the time-pickoff circuit.

From Figure 15, two conclusions can be clearly stated as follows: firstly, k adjusted from 0.2 to 0.6 can be applied in the time-pickoff circuit for ranging measurements, which is shown in block 3 (red area) in Figure 15, because when k is beyond the range of 0.2~0.6, the ranging precision deteriorates. This choice criterion also can be deduced from the simulation results as shown in Figure 9. Secondly, the optimal k and $t_{d}$ should be selected according to the range performance of the minimum pulse amplitude, such as the left one of the three bars in each group data in Figure 15.

## 6. Conclusions

This paper proposed determination criteria for the ranging circuit parameters of LiDAR systems. From the simulation and experimental results, two circuit parameters (the attenuation fraction k and the delay time $t_{d}$) should be selected according to the ranging performance of the minimum pulse amplitude. The attenuation fraction (k) should be adjusted between 0.2 and 0.6. The delay time $t_{d}$ should be selected to satisfy the “minimal selected-range” of the ranging precision curve as varied with the k. The parameters selection might vary with changing chips and circuit designs, but the choice criteria follow the same physical rule.

## Figures and Tables

**Table 1:** Grading of the PGA gain. The PGA gain is adjusted to concentrate the input pulse amplitude (0.05–3 V) into a limited range (1–1.5 V).

**Pulse amplitude range (V)**	0.05–0.25	0.25–0.42	0.42–0.70	0.70–1.18	1.18–1.98	1.98–2.5	2.5–3
**Gain of PGA (dB)**	20	15.5	11	6.5	2	0	−2
